# The Use of Chloroform and Aconite in Neuralgia

**Published:** 1860-02

**Authors:** 


### THE USE OF CHLOROFORM AND ACONITE IN NEURALGIA.

In a letter to the *Med. Times and Gazette*, Dr. Gueneau de Mussy writes:—

"For more than three years I have prescribed it in neuralgia of different regions; but here I want chiefly to allude to the most common and severe of neuralgic pains—the facial neuralgia, in which I have derived from the above-named remedy sometimes a complete and permanent cure, and always an almost immediate and considerable relief. In such cases I apply it directly to the gums of the side affected, where numerous divisions of the fifth nerve are most superficial. When the neuralgia is idiopathic, *i. e.* unconnected apparently with any other disease, the formula is:—2 parts of sp. of wine, or eau-de-Colonge, 1 of chloroform, and 1 of tincture of aconite; the finger covered with a piece of lint or soft thick linen, is dipped in the mixture and rubbed gently against the gum for a few minutes. I do not use a sponge, because it would take up too much of the liquid, which by pressure might drop into the mouth.

"When the pain is connected with some organic disease, as a deranged tooth, chronic inflammation of the gums, or of the sockets, or, as I have observed, superficial necrosis of the bone, I have found the tincture of iodine a very beneficial substitute for spirit of wine in the formula above.

"The infra-orbitary branch being the most commonly affected, it is chiefly in neuralgia in this part the application has been successful; but by no means exclusively so; it answers very well in pain of the lower branch, and I have observed some very severe cases of supra-orbitary neuralgia, in which the same application has been attended with an equally satisfactory result.

"This shows, moreover, that the sedative agent may produce its effect, as the irritating one so frequently does, on a part distant from the spot on which it was applied in the same, and even in a different branch of a nerve."

